# ‘It helps my anxiety because I’m managing my breathlessness’: a qualitative exploration of anxiety and breathlessness in patients with advanced chronic respiratory disease receiving specialist palliative care

**DOI:** 10.1136/bmjopen-2025-112993

**Published:** 2026-05-03

**Authors:** Lucy Bleazard, Kate Walker, Sue Ashton, Christina Faull

**Affiliations:** 1 LOROS Hospice, Leicester, UK; 2 University of Leicester, Leicester, UK; 3 Northamptonshire Healthcare NHS Foundation Trust, Northampton, UK

**Keywords:** PALLIATIVE CARE, Adult palliative care, RESPIRATORY MEDICINE (see Thoracic Medicine)

## Abstract

**Abstract:**

**Objectives:**

To explore the lived experiences of anxiety related to breathlessness in patients who are receiving specialist palliative care for advanced chronic respiratory disease (CRD), such as chronic obstructive pulmonary disease and interstitial lung diseases.

**Design:**

This qualitative exploration formed part of a mixed-methods feasibility study of a novel intervention. Participants receiving specialist palliative care for CRD engaged in semi-structured interviews. Data were analysed using thematic analysis.

**Setting:**

Two hospice sites in a single UK region.

**Participants:**

11 participants were included in the analysis (7 male participants and 4 female participants with an age range of 49–75 years). Ethnicities were self-reported as white British (n=8) and Asian/British Asian (n=3).

**Results:**

Three key organising themes emerged: (1) Understanding my breathlessness—participants described their breathlessness as progressive, frightening and restrictive. (2) Understanding my anxiety—described as emotional distress with a profound physical component, often compounded by external life stressors. (3) Vicious circle interlinks breathlessness and anxiety—a circular bi-directional relationship where anxiety could not be separated from breathlessness. Participants often employed non-pharmacological strategies to manage both their anxiety and breathlessness interdependently.

**Conclusions:**

Our findings provide insight into the intricate relationship between anxiety and breathlessness in patients with a range of CRD. There is an ongoing need for holistic support for patients with anxiety related to breathlessness in non-malignant conditions.

**STRENGTHS AND LIMITATIONS OF THIS STUDY**

- This qualitative study includes patients with a range of advanced chronic respiratory diseases from two separate UK hospice sites to expand the breadth of perspectives captured, with a specific focus on the experience of anxiety.
- Thematic analysis was conducted by two researchers from differing professional backgrounds to enhance analytical rigour.
- The sample size is modest and limited to a single UK region, which limits transferability.
- The use of a single interview, as opposed to serial interviews, may limit participants’ recall of previous experiences of their anxiety and breathlessness.

## Introduction

 Chronic respiratory disease (CRD) is one of the leading causes of death worldwide,^1 2^ encompassing a range of conditions including chronic obstructive pulmonary disease (COPD) and interstitial lung diseases (ILD). CRD is characterised by persistent, progressive breathlessness.^3^,^5^ This debilitating physical symptom is often the primary focus of symptom management in CRD, and while this is both necessary and justified, many patients with CRD will experience substantial psychological distress alongside their breathlessness, which is frequently under-recognised and insufficiently addressed.^6^

Anxiety constitutes a significant component of this psychological burden. Up to one-third of patients with COPD will meet the criteria for diagnosis with generalised anxiety disorder,^7^ with comparable estimates for ILDs.^8^ The relationship between anxiety and the physiological experience of breathlessness in CRD is complex, as feelings of anxiety amplify the perception of breathlessness and vice versa.^9^ As a result, the management of anxiety related to breathlessness in advanced CRD presents a significant clinical challenge.

Holistic multidisciplinary breathlessness services, such as those offered by specialist palliative care or as part of some respiratory services, have demonstrated efficacy in supporting patients to manage their breathlessness.^10^,^14^ However, there has been little exploration of how patients using these services experience and make sense of their anxiety related to their breathlessness.

This aim of this analysis was to explore these lived experiences for patients using specialist palliative care (SPC) for a range of CRD diagnoses. This work forms part of a wider feasibility study exploring the potential use of neuromodulation in the management of anxiety related to breathlessness.

## Methods

### Overview

This work formed part of a 12-week mixed-methods interventional feasibility study, which included a single semi-structured interview with participants at the end of their involvement. We report here analysis of those interviews with respect to participants experiences of anxiety related to breathlessness. Participants were recruited at two specialist palliative care services, both of which provide multidisciplinary outpatient services for patients with advanced CRD. This study has been reported in accordance with the Standards for Reporting Qualitative Research (SRQR) reporting checklist.^15^

### Participants

Participants were receiving SPC at the recruiting hospice site for CRD of any aetiology. Diagnoses were recorded as documented in their electronic patient record. Eligibility criteria included an Integrated Palliative Care Outcome Scale (IPOS)^16^ score of >2 for both ‘shortness of breath’ and ‘anxious/worried’ domains, which had been routinely administered as part of clinical care. All patients attending SPC outpatient breathlessness services were screened for eligibility, and a purposive sampling strategy was used to ensure a representation across a range of CRD. This qualitative analysis included only participants allocated to the intervention (Alpha-Stim) arm of the wider feasibility study; all participants in this group (n=11) were invited to interview and all consented.

### Recruitment

Members of the patients’ usual clinical team introduced the study to those identified through screening. Individuals who expressed an interest in the study were provided with a written participant information sheet and contacted by a member of the research team to confirm eligibility and willingness to proceed. Written informed consent was obtained from eligible patients prior to enrolment.

### Data collection

Participants were invited to participate in an in-depth semi-structured telephone interview with an experienced qualitative researcher towards the end of the 12-week study period to explore their lived experiences of anxiety and breathlessness. The interviewer (KW) was a senior qualitative researcher primarily working within mental health settings. The topic guide was developed to explore experiences of anxiety and breathlessness separately, including descriptions of sensations experienced, usual triggers, alleviating and perpetuating factors and management strategies. The topic guide also included a section on the use of the novel intervention which is not included in this analysis. A brief introduction to the purpose of the interview was provided prior to beginning, as well as reassurance to participants that they may take frequent breaks if they became breathless during the interview. The topic guide is presented in the online supplemental file 1. There was no prior established relationship between the researcher conducting the interviews and the participants. Interviews were audio-recorded onto an encrypted recorder and interview lengths ranged from 28.25 to 38.59 min (M_minutes_=32.59, SD=3.54). Audio files were transcribed verbatim and anonymised at the point of transcription.

### Data analysis

Interview data were analysed using thematic analysis as a means of organising the data into patterns and themes^17 18^ and to highlight differences and similarities between participants’ accounts.^19^ Thematic analysis allows researchers to take a structured approach to managing and analysing the data, and so is suitable for identifying patterns across and within the interviews in relation to the attitudes, beliefs, perceptions and experiences of the participants.^19 20^ Themes were developed using an inductive approach at the semantic level, therefore based on the explicit or surface meanings of the data.^18^ Data analysis followed the six-step framework that is consistently used for thematic analysis.^17 18^

The primary analyst (KW) was an experienced qualitative researcher with no prior involvement in palliative care research, allowing for an external perspective on the data. The secondary analyst (LB) was an early career researcher and clinical trainee in palliative medicine, bringing insight into the palliative care context. This combination of perspectives allowed for a balanced interpretation of the data, and reflexive discussions between the two analysts were used throughout the analysis process. Neither analyst had any prior clinical or research involvement with any participant. Analytical rigour was further enhanced through investigator triangulation, with a third researcher (CF) reviewing the interview transcripts and the development of themes to provide an independent perspective on the analysis.

Verbatim (anonymised) quotes were identified and included to promote verifiability and trustworthiness of the analysis.^21^ To ensure that the findings were the experiences of those interviewed, strategies advocated by Shenton^22^ were implemented to enable credibility and confirmability, including reflexive analytical practice and systematic audit of coding decisions. To enhance transparency and demonstrate the analytical process, an example of the progression from raw data to meaning units, initial codes, subthemes and organising themes is presented in table 1. This illustrates how participants’ accounts were systematically interpreted and synthesised during inductive thematic analysis. Trustworthiness was further supported through comparison of accounts across participants, enabling convergence and divergence of perspectives to be examined within the thematic analysis. Analysis was completed once thematic saturation was reached and no new themes were perceived to emerge from the data. As this analysis was performed as part of a larger feasibility study, thematic saturation did not affect further recruitment to the study. However, any further interviews focused on the novel intervention and experience of being in the study, not the participants’ experience of anxiety and breathlessness.

**Table 1:** Example of the analytical process from raw data to organising themes

Raw data (verbatim quote)	Meaning unit	Initial code	Subtheme	Organising theme
“Throughout those 10 years, it has slowly gotten worse… now it is probably the worst it’s ever been.” (P5)	Breathlessness worsening over time	Progressive decline in symptoms	Something progressive, that deteriorates over time	Understanding my breathlessness
“I could go out shopping on my own… whereas now, I can’t do that anymore.” (P7)	Loss of independence due to breathlessness	Functional decline	Restricts me, unable to do day-to-day activities	Understanding my breathlessness
“Sometimes you feel that this is your last breath.” (P2)	Breathlessness experienced as life-threatening	Fear of suffocation	A state that induces fear, panic and feeling out of control	Understanding my breathlessness
“My heart starts racing… I feel hot.” (P5)	Physical panic response to anxiety	Physiological anxiety symptoms	Associated with physiological reactions	Understanding my anxiety
“It’s like a panic feeling… I can’t concentrate.” (P5)	Emotional experience of anxiety	Panic and fear	A state that induces panic, fear and deep worry	Understanding my anxiety
“I manage my anxiety by managing my breathlessness.” (P4)	Anxiety reduced when breathlessness controlled	Breathlessness drives anxiety	Manage breathlessness to manage anxiety	Understanding my anxiety
“You’re breathless, so that makes you anxious… then anxious makes you more breathless.” (P2)	Bidirectional escalation	Symptom reinforcement loop	—	Vicious circle interlinks breathlessness and anxiety
“I do mindfulness and breathing exercises.” (P1)	Use of coping techniques	Self-regulation strategies	Breathing exercises, distraction and relaxation techniques	Understanding my breathlessness
“Try to build positive feelings all the time.” (P10)	Cognitive coping for anxiety	Positive reframing	Actioning distraction, relaxation and positive mindset	Understanding my anxiety

### Patient and public involvement

Patient and public involvement (PPI) work was carried out at the inception of this project with patients living with advanced chronic respiratory disease receiving specialist palliative care, as well as members of the public interested in research via the hospice’s PPI research consultee group. PPI consultees advised the study team on several aspects of the work, including the acceptability of the research question, the development of study documents, methods of data collection and the recruitment strategy.

## Results

The 11 participants who completed an interview comprised 7 male participants and 4 female participants with an age range of 49–75 years. Their self-reported ethnicities were white British (n=8) and Asian/British Asian (n=3). The primary diagnoses were reported as COPD or emphysema (n=6), ILD (unspecified type) (n=2), idiopathic pulmonary fibrosis (n=1), sarcoidosis (n=1) and bronchiectasis (n=1). All participants reported clinically significant levels of breathlessness and anxiety at baseline, as indicated by IPOS scores >2 in both domains. None of our participants had a formal diagnosis of anxiety disorder.

**Figure 1:**
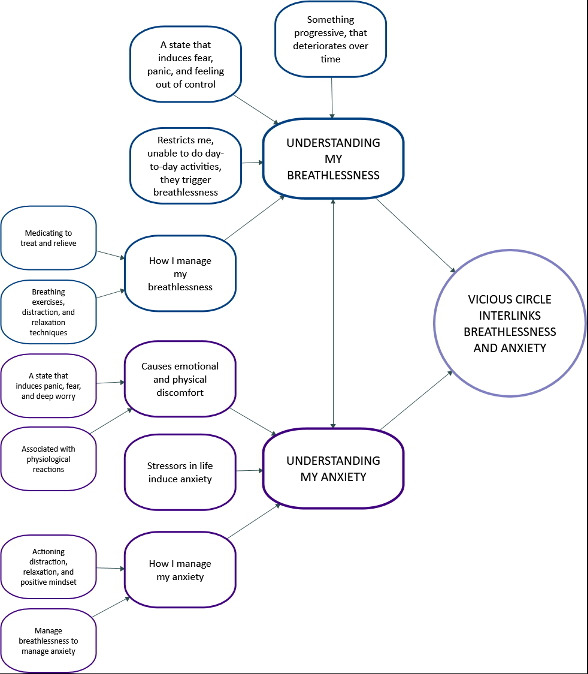
Themes and subthemes of patients’ experience of anxiety and breathlessness.

Three organising themes were developed, presented in figure 1: (1) Understanding my breathlessness; (2) Understanding my anxiety; and (3) Vicious circle interlinks breathlessness and anxiety. The first two organising themes are represented by several subthemes, while the third organising theme was developed as a standalone theme. Together, these themes capture how participants made sense of their breathlessness and anxiety not as isolated symptoms, but as evolving, embodied experiences that shaped daily life, emotional well-being and self-management.

### Understanding my breathlessness

This organising theme reflects how participants conceptualised breathlessness as a progressive, frightening and function-limiting experience, alongside the strategies they used to regain control. Breathlessness was not described merely as a physical symptom but as a persistent presence that structured participants’ routines, expectations and emotional responses.

#### Something progressive, that deteriorates over time

Participants consistently framed breathlessness as evidence of irreversible decline. Rather than episodic discomfort, it was experienced as a gradual but relentless worsening that signalled loss of health, independence and normality. This progressive trajectory shaped how participants understood their illness and future. P5 described a slow but continual deterioration:

P5: So, throughout those 10 years, it has just slowly gotten worse. So now it is probably the worst it’s always, it’s ever been. But it, as time’s getting on, it is getting worse and worse.

Within the narratives, there was reference by several to this being something that was ‘deteriorating’ over time and as such was preventing them from doing things they previously were able to do. P7 explicitly linked breathlessness to shrinking independence:

P7: Deterioration in my condition. It is simply the deterioration in my condition. I could go out shopping on my own, just with my oxygen. I was driving. I would go out on my own as well. Whereas now, I can’t do that anymore. It’s the, the speed of it deteriorating.

Across accounts, participants recognised that improvement was unlikely, and management rather than recovery became the dominant goal.

P6: There is nothing that will improve it, but I’ve just got to try and manage it. It made me realise just how bad things could be. Now looking at it, well I am breathless all day, you know, I am never going to get any better.

This acceptance of permanence appeared to underpin emotional distress and contributed to heightened vigilance around symptom changes.

#### A state that induces fear, panic and feeling out of control

Breathlessness was universally associated with intense fear and panic, often described in existential terms relating to suffocation or death. Participants portrayed breathlessness as qualitatively different from ordinary exertional breathlessness, characterised instead by a loss of bodily control and overwhelming threat.

P2: It’s scary. Sometimes you feel that this is your last breath.

Some participants offered illustrative analogies and metaphors were frequently used to communicate the severity and abnormality of the sensation, and in doing so offered a rich description of what this experience is really like for them.

P1: Scary. It’s like trying to climb a mountain with a plastic bag over your head.

P7: So, it’s like permanently having a bag over your head or a scarf over your mouth and nose.

P11: So that feeling of your drowning almost but drowning in air.

These metaphors conveyed both restriction and terror, highlighting how breathlessness was perceived as life-threatening rather than merely uncomfortable. Physiological panic responses such as racing heart, overheating, palpitations often accompanied episodes, reinforcing the sense of crisis:

P8: Then heart starts racing… I’m really burning hot.

Together, these descriptions illustrate breathlessness as a multi-sensory experience combining physical distress and acute emotional threat.

#### Restricts me, unable to do day-to-day activities, they trigger breathlessness

All participants identified that doing certain activities triggers their breathlessness. Exertion of any type was felt to be a trigger, breathlessness transformed routine activities into sources of risk and anxiety, fostering dependence.

P5: If I exert myself in any way, the stairs … even now I’m talking to you, I’m getting slightly out of breath. So walking, any physical activity, bending down, anything at all causes me to become very breathless.

This all restricts the individual and means they are unable to engage in their usual activities of daily living. Participants reflected that these were activities most people would take for granted. These included washing and dressing, playing with their children and housework:

P5: Playing with your children, that probably had the most impact on me, it makes you realise that yes, this is actually impacting a lot of my lifestyle now, practically all of it.

This functional restriction appeared to reinforce awareness of illness progression and contributed to emotional distress, frustration and social withdrawal.

#### How I manage my breathlessness

Participants manage their breathlessness in a variety of ways. This emerges through two subthemes, one relating to pharmacological management and one to non-pharmacological strategies.

#### Medicating to treat and relieve

Many participants use pharmacological management to control their breathlessness. Regular medication, primarily a variety of inhaled medication and oral mucolytics, was described by most participants. This was perceived as controlling their underlying disease, and therefore managing their breathlessness.

P8: I have regular medication - inhalers, I have tablets as well. With the tablets they are for like the phlegm and things like that. The only thing I have got for the COPD is the Trimbow inhaler, that is the only medication there really is for it.

The other use of medication was to relieve their breathlessness at times of escalation, by taking as-required inhalers or oral opioids. One participant described using supplemental oxygen to control their breathlessness when it became overwhelming.

P3: Taking oxygen obviously makes it [episode of breathlessness] better. Nights can be a bit of a problem… I just take some morphine or pull on me inhaler or something like that. I take the Ventolin whenever I need it.

These were often described pragmatically, as necessary tools rather than cures.

#### Breathing exercises, distraction and relaxation techniques

Non-pharmacological strategies were framed as empowering and immediately accessible and employed these regularly, including mindfulness, grounding and breathing exercises.

P1: So, I do my mindfulness and grounding techniques. So, it’s the 5,4,3,2,1. I also do a lot of breathing exercises where I breathe in for four and exhale for five. I have a window that’s an oblong and I breathe in and out on the ups and downs and the sides of the window and stuff.

Similarly, some participants also discussed that techniques they used were around relaxation, feeling calmer or trying to sleep, described rehearsed routines that provided psychological reassurance as much as physical relief.

P2: Relaxing. Definitely trying to relax. And have a sleep if you can. I have to have one every afternoon just for an hour, or even if you don’t go sleep, just go and lay on the bed.

Many participants employed distraction techniques to divert their attention from their breathlessness, including reading, watching television or using their mobile phone.

P5: I use a form of distraction, not be thinking about, you know, a) my cough and b) my breathing. So, I use a form of distraction. I always have my phone with me, so the main thing I'll do just go on my phone and start reading the news, or go on Twitter or, or something, that will take my mind off, what’s actually happening to me.

Some participants also described using airflow strategies, such as handheld or electric fans, to relieve breathlessness and reduce anxiety.

P10: I just as I say, sit down, do my breathing exercise and use a small fan… that gets back my breathlessness.

Collectively, these strategies reflect attempts to interrupt panic, regain bodily control and cope with the unpredictability of symptoms.

### Understanding my anxiety

This organising theme captures anxiety as both an emotional state and a physical experience, often experienced as pervasive rather than episodic. Participants conceptualised anxiety as rooted in illness but amplified by broader life stressors. It is represented by three individual themes.

#### Causes emotional and physical discomfort

Participants experience anxiety as a sensation of discomfort, both emotional and physical. This is presented as two subthemes to reflect each of these domains.

#### A state that induces panic, fear and deep worry

Anxiety was frequently described using the same language as breathlessness, for example, panic, fear and dread, suggesting experiential overlap:

P5: It’s like a panic feeling, and I, it, I feel a bit zoned out as well. And I can’t concentrate on anything around me, and, yeah, so this, it’s the feeling panicked.

P4: The anxiety is like a feeling of fear, it’s like fear. I mean I went to the toilet one time, and I got off the toilet and just fell, I collapsed. That really got me, it really did honestly.

Many participants described a constant underlying fearfulness that permeated daily life:

P8: It is like when you are anxious isn’t it. I go shaky and stuff. It is like I don’t know; I am just scared all the time.

#### Associated with physiological reactions

Alongside the distressing emotions associated with their anxiety, participants also discussed having physical reaction to their anxiety. Examples participants described included feeling their heart rate increasing:

P2: Anxiety is when your heart rate going. And you sort of hyperventilated. Well, I can feel my heartbeat and it probably isn’t and it feels like it’s going fast you know; sweating palms and feeling hot.

P5: I feel panicked a little bit, and my heart starts raising. I, my palms start sweating. I feel hot. Those are the physical feelings that I get; and nausea.

P6: Yes, it is dread. It is fear. You get anxious. You, it makes me, it even makes me feel sick and I start heaving and I’m not being sick. It really makes me start to retch like.

This physicalising of anxiety appeared to blur boundaries between psychological and respiratory symptoms.

#### Stressors in life induce anxiety

Participants described having a baseline ‘general anxiety’ because of their diagnosis, prognosis and knowledge of their disease. Different stressors would then occur during day-to-day life that would build on and worsen their baseline anxiety.

P11: I don’t know what came first, the chicken or the egg. I, I’m not sure. My anxiety is tied to a degree to my diagnosis. It’s tied to a lot of other things other than my breathlessness.

A wide range of events were perceived as being stressors, from taking their child to college to family bereavements. The time of year was frequently cited, with the arrival of winter or the Christmas period often worsening their feelings of anxiety.

P1: Again, you’ve caught me at the wrong time, with anxiety because I’ve had so much going on. It’s Christmas and you know, sort of being with family and everything.

This suggests anxiety was not solely reactive to breathlessness but embedded within broader psychosocial contexts.

#### How I manage my anxiety

This theme comprises two subthemes that are very much interlinked. The first relates to strategies implemented when participants are experiencing their ‘general activity’. The second relates to the management of their breathlessness when this is seen as the cause of their anxiety; thereby if breathlessness is managed, the associated anxiety will be too.

#### Actioning distraction, relaxation and positive mindset

When participants feel generally anxious, they implement strategies, tools and techniques such as distraction and relaxation. The strategies described were often similar to those used to manage exacerbations of breathlessness.

P2: Just try to keep as busy as I can. Keep me mind occupied, either reading or games or television or something. Try not to think about it too much you know. Mind over matter I suppose.

Many participants described dealing with their anxiety by attempting to change their thinking, to adopt a positive mindset, or to create ‘positive energy’, which was notably absent when discussing how to manage their breathlessness.

P10: It’s the thought process you know, all the negative thinking which create anxiety and I, try to build positive feelings all the time. I try to counter my activity with positive energy. I tried to feel positive energy within me.

#### Manage breathlessness to manage anxiety

Participants often described dealing with their breathlessness as a method of dealing with their anxiety, acknowledging the link between the two. This leads us into our final organising theme.

P4: I have just got to sit down and start breathing, slowly. So, because it’s related to my breathlessness, I manage my anxiety by managing my breathlessness. It takes the pressure off – like a boiling kettle! I guess it helps with my anxiety because I’m managing my breathlessness.

For many, anxiety was viewed as secondary to breathlessness, something that would resolve if breathing could be controlled.

### Vicious circle interlinks breathlessness and anxiety

This final organising theme draws together the experiences of participants of their anxiety and their breathlessness, as many discuss how these are inextricably linked to one another. Participants experience breathlessness causing anxiety, but then anxiety causes breathlessness—a bi-directional causal relationship between anxiety and breathlessness, mediated by fear and panic of both experiences. Participants described this as part of their everyday life, not just in times of acute exacerbation.

P2: I think it’s just terrifying when you can’t breathe, so it just forces anxiety….so I breathe, and I can’t really breathe, and I feel like I am suffocating. So, it’s definitely this anxiety comes when I’m actually feeling breathless, and then being anxious makes me more breathless.

Participants explicitly named this as a ‘vicious cycle’, describing it as part of everyday life rather than only acute episodes. Participants feel that they keep going round in a continual circle of breathlessness-anxiety-breathlessness. Management techniques can help to reduce the sensations or potentially, although temporarily, break the circle.

P9: Because you can’t breathe, you do get anxious, you get panicky. So, you’re breathless, so that makes you anxious, and then as you are anxious, that then makes you more breathless, as it were, it’s a vicious cycle.

Across themes, participants constructed breathlessness and anxiety as intertwined embodied experiences characterised by fear, loss of control and progressive restriction, managed through overlapping pharmacological and self-regulatory strategies aimed at restoring stability and interrupting cyclical escalation.

## Discussion

This qualitative study explores how patients receiving specialist palliative care experience and make sense of their anxiety and its complex relationship with breathlessness. Our participants reported a bi-directional interplay between these symptoms, often conceptualised in terms similar to the ‘dyspnoea-anxiety-dyspnoea cycle’ described by Bailey,^9^ where breathlessness provokes fear, leading to worsening symptom perception and avoidance behaviours. Participants’ experiences can also be usefully interpreted through the ‘Breathing, Thinking, Functioning Model’.^23^ This model provides a framework for understanding the interacting components of breathlessness, encompassing physiological breathing patterns, cognitive and emotional responses and functional behaviours. Within this framework, our findings illustrate how these domains might interact dynamically, with anxiety influencing activity and social participation, and vice versa.

The novel contribution of this study in lies in our participants’ differentiation between anxiety directly triggered by breathlessness episodes and a more pervasive psychological anxiety linked to living with a progressive, life-limiting illness with an unpredictable trajectory. A recent systematic review by Bolton *et al* exploring existential distress among patients with advanced COPD identified that patients often feel they exist within a ‘liminal space’, an ambiguous state where they are neither able to live a healthy life nor actively dying.^24^ Our findings extend this concept by showing how attempts to sustain everyday activities become a site of tension, simultaneously increasing breathlessness and provoking psychological distress by reinforcing awareness of irreversible change.^25 26^ Although many of our participants reported symptoms consistent with generalised anxiety disorder, none had received a formal diagnosis. This reflects the existing literature indicating patients’ psychological distress is often highly prevalent and consistent with formal psychiatric disorders, yet often under-recognised and under-treated.^7 8^

Exploring the lived experiences of people with advanced CRD is crucial for informing the design of interventions that are responsive to their needs. Research over recent decades has demonstrated how dedicated breathlessness intervention services (BIS) can improve patients’ quality of life and reduce distress related to breathlessness.^11 14 27 28^ Underpinned by a holistic, patient-centred approach, BIS combine non-pharmacological and pharmacological techniques to support patients.^29^ Our findings align with this literature, with participants describing the use of a range of self-management strategies, to effectively self-manage their symptoms regardless of whether anxiety or breathlessness was perceived to be the primary symptom. Whereas previous literature has described patients with advanced CRD as more inclined to rely on hospital intervention rather than self-management,^30^ our participants were able to explain how these techniques empower them to regain a sense of control over their symptoms. This highlights the potential of non-pharmacological techniques and the holistic patient-centred approach, such as those offered in BIS, to promote autonomy and resilience in the face of symptomatic crises.

The findings reinforce the holistic approach offered by multidisciplinary BIS offered by a range of services. There is a clear need to recognise the significant psychological burden of living with an advanced CRD, and clinicians should consider specifically inquiring with their patients regarding symptoms of anxiety or panic disorders. Multidisciplinary approaches integrating psychological and non-pharmacological strategies alongside physical symptom management may be beneficial. Future research may wish to explore how experiences of anxiety and breathlessness evolve longitudinally throughout the disease trajectory and potential barriers to patients with advanced CRD accessing specialist breathlessness services.

This study encompasses patients with a range of CRD diagnoses rather than focusing on a single disease. All participants were identified as having high levels of anxiety and breathlessness at baseline using the IPOS, which is a widely used tool in the palliative care setting. The sample size is small yet reasonable for a qualitative study using thematic analysis, and thematic saturation was reached for this analysis once no new themes were perceived to be emerging from the data. The participants included in this analysis were recruited from two sites in a single region, which may limit transferability and constrain triangulation across settings, despite the use of strategies to enhance analytical trustworthiness.

## Conclusion

This study provides valuable insight into the intricate relationship between anxiety and breathlessness in patients with a range of CRD accessing specialist palliative care services. Our findings emphasise that anxiety cannot be separated from breathlessness, and there is an ongoing need for holistic support for patients with anxiety related to breathlessness in non-malignant conditions.

## Supplementary material

10.1136/bmjopen-2025-112993 online supplemental file 1

## Data Availability

No data are available.
