# Supplementary material for: ‘It helps my anxiety because I’m managing my breathlessness’: a qualitative exploration of anxiety and breathlessness in patients with advanced chronic respiratory disease receiving specialist palliative care
Source: BMJ Open. 2026 May 3;16(4):e112993. doi: 10.1136/bmjopen-2025-112993 (PMC13141109; doi:10.1136/bmjopen-2025-112993)
Supplement: online supplemental file 1 [file bmjopen-16-4-s001.docx]

| **A scoping study to evaluate the potential of an electronic medical device to treat anxiety in people who experience breathlessness.** |
| --- |
| Topic Guide v1.0 - for **intervention group** participants |

Instructions for interviewer:

*The aim of this interview is to explore the lived experience of the participant’s feelings of anxiety and breathlessness over the study period, as well as explore their views on the Alpha-Stim AID device and their experience of being involved in this study. This document should serve as a guide only on which the conversation should be built. Please ensure you have blank copies of the participant questionnaires with you in order to facilitate the last part of the interview.*

*Please be aware, this patient group may find talking for extended periods of time difficult if they are experiencing severe breathlessness. Ensure the participant is in a comfortable space before starting and offer breaks as often as necessary. If the patient is using an oxygen concentrator this may create additional noise, so please test that the audio recording is audible before starting. If the interview has to be terminated due to breathlessness, it can be completed at a later date if appropriate and if the participant agrees to this.*

**Introduction**

Today I would like to speak to you about your experiences of anxiety and breathlessness, as well as your experiences of being a part of this study and using the Alpha-Stim AID device. There are no right or wrong answers, I just want to hear about your experiences and opinions. As you know, this study is a small initial study to help us understand how to design a larger study on this topic in the future, so I’d like to hear about your experience of being involved in this study and any feedback you have.

All the interviews will be pseudonymised afterwards, meaning all personal details and names will be removed, so you will not be identified. You do not have to answer all of the questions. If you’re not comfortable answering a question, we can skip it and move on to the next question.

The interview today should last around 45 minutes to an hour. If you need to take a break at any time, please let me know. You can take as many breaks as you need.

Do you have any questions before we start?

For our data collection, we will be audio recording this interview. I will start the audio recorder now.

[*Begin audio recording. State participant ID number and date.*]

**Alpha-Stim AID device**

Can I ask, what is your respiratory condition or illness please?

[*patients will have a diagnosis of COPD, ILD, or pulmonary fibrosis of any cause*]

Tell me about your experience of using the Alpha-Stim AID device.

[*give no cues during initial narrative, then prompt as needed:*]

- Tell me about a typical session – what time of day did you use it? Did you use it for the full hour? What did you do whilst using it?
- How did you feel whilst using the Alpha-Stim AID? What about directly afterwards?
- Do you think it helped you feel less anxious?
- Did you experience any side effects? If yes, did we adjust your treatment regime? What were they? When did they occur?
- Were there any days you didn’t manage to complete a session? If yes, please tell me about why that was.
- Would you consider using the device again if you were offered it?
- Did you feel you had enough support and instruction on how to use the device?

**Breathlessness**

To start us off, please tell me about your breathlessness.

[*give no cues during initial narrative, then prompt:*]

- How long have you felt breathless for?
- What does it feel like to be breathless?
- What makes your breathlessness worse?
- What makes your breathlessness better?

Thinking back over the last 12 weeks, tell me how your breathlessness has been.

[*prompts:]*

- Has it got better, worse, or stayed the same?
- Why do you think that is?

Tell me what you do when you feel breathless. How do you manage it?

[*prompts:*]

- Do you use any medicines?
- Do you use any techniques that don’t involve medicines?
- Do you feel able to manage your breathlessness?
- Did you attend the Help With Breathlessness service during these 12 weeks?

[*if yes, tell me about your experience. What happened at the appointment? Did it help?*]

**Anxiety**

As you know, this study was also exploring anxiety levels in people who feel breathlessness.

What does ‘anxiety’ mean to you?

[*this is an invitation to open up the conversation to discuss anxiety. Give no cues during the initial narrative, then begin to prompt:*]

- Please tell me about the feelings of anxiety that you experience.
- Do you think feeling anxious is related to feeling breathlessness? Why?

Thinking back over the last 12 weeks, tell me about your levels of anxiety.

[*prompts:]*

- Has it got better, worse, or stayed the same?
- Why do you think that is?

Tell me what you do when you feel anxious. How do you manage it?

[*prompts:*]

- Do you use any medicines?
- Do you use any techniques that don’t involve medicines?
- Do you feel able to manage your anxiety?

**Diaries**

During this study, we asked you to keep a diary and give your breathlessness and anxiety a score every day, at best, at worst, and on average.

Tell me how you found completing the diary.

[*prompts:*]

- Did you complete it every day?
- Did you find it easy or hard to give a score every day?
- What made you give a high score? A low score? [*ask for both breathlessness and anxiety*]

[*If the participant still has their daily diary, at this point ask them to show you a day in the diary and talk through the scores they gave that day.*]

**Questionnaires**

We asked you to complete several questionnaires throughout the study.

[*you should have blank copies of each of the questionnaires in order to facilitate discussion and remind the participants of the questionnaires they have been asked to complete, as there are several*]

Tell me about how you found completing the questionnaires.

[*prompts:*]

- Did you find them easy or hard to complete?
- Which do you think best represents your breathlessness? [*ask again for anxiety*]
- Which one did you feel was the most relevant to you?
- Were there any that you didn’t find relevant to you?
- Was there anything you felt the questionnaires didn’t cover?
- Were any of the questionnaires upsetting to complete?

**Study experience**

Tell me about your experience of being in this study.

[*prompts:*]

- Did you enjoy being part of this study?
- What do you think we could improve for the future?
- Did you have all the information you needed at the start of the study?
- Would you participate in other studies in the future?

**Close of interview**

That concludes my questions for today. Is there anything else you would like to add to what we’ve already discussed?

Thank you again for your participation in this study. This is the end of the interview.

[*stop audio recording*]
