# Supplementary material for: ‘It helps my anxiety because I’m managing my breathlessness’: a qualitative exploration of anxiety and breathlessness in patients with advanced chronic respiratory disease receiving specialist palliative care
Source: BMJ Open. 2026 May 3;16(4):e112993. doi: 10.1136/bmjopen-2025-112993 (PMC13141109; doi:10.1136/bmjopen-2025-112993)
Supplement: Supplementary file 2 [file bmjopen-2025-112993.reviewer_comments.pdf]

PEER REVIEW HISTORY

BMJ Open publishes all reviews undertaken for accepted manuscripts. Reviewers are asked to complete a checklist review form and are provided with free text boxes to elaborate on their assessment. These free text comments are reproduced below.

ARTICLE DETAILS

Title (Provisional)

'It helps my anxiety because I'm managing my breathlessness': a qualitative exploration of anxiety and breathlessness in patients with advanced chronic respiratory disease receiving specialist palliative care

Authors

Bleazard, Lucy; Walker, Kate; Ashton, Sue; Faull, Christina

VERSION 1 - REVIEW

**Reviewer**	**1**
**Name**	**Smith, Tracy**
**Affiliation**	**Westmead Hospital, Department of Respiratory and Sleep Medicine**
**Date**	**04-Jan-2026**
**COI**	**None**

The authors have undertaken a qualitative study of breathlessness and anxiety in patients with chronic respiratory disease. The methods are adequate to the research question and the analysis seems to be superficial, but reasonable.

However, the authors have not sufficiently engaged with the deep and long history of research in this area which goes back at least 40 years. The discussion, in particular, is underdeveloped and needs more work. Much of the discussion is a restatement of the results. Other than a brief mention of a paper from Bailey et al, the authors have not situated their paper within the broader literature. Perhaps due to this, it is not clear what the unique and new offering of this paper is to the literature. I think there may be some insights in the results about patients' understanding of the difference between breathlessness-induced anxiety and psychologically-based anxiety which may form the basis of a unique contribution. The experience of breathlessness and anxiety have been explored previously, though there is perhaps room to further explore this. Additionally, the discussion is missing at least some mention of implications for clinicians/ policy makers, as well as implications for future research.

Hence I recommend a major rewrite. The authors need to show evidence of an extensive review of the literature and develop and argument about the unique offering that this piece adds in the context of the available literature.

This may require:

Some specific comments:

• Note should be made of the SRQR reporting standards in the methods

• Researchers characteristics are insufficiently described in the manuscript. Mention should be made at least of their discipline and other characteristics. Assuming the primary and secondary analysts are authors their initials should be included on page 6.

• Authors note that techniques to improve trustworthiness are discussed on page 5. I cannot see this discussion on either page 5 or 6, but note used of techniques to enable credibility and confirmability on page 7. These contribute to trustworthiness but are not sufficient. There is no mention in particular of triangulation.

• Trustworthiness does not appear to be specifically discussed in the limitations.

• The conclusions drawn on page 15 lines 44 – 50 regarding suitability of a unified approach to care in breathlessness clinics is an over reach compared to the data presented and requires substantial revision.

**Reviewer**	**2**
**Name**	**Christensen, Vivi Lycke**
**Affiliation**	**University of South-Eastern Norway, Department of Nursing and Health Sciences**
**Date**	**05-Jan-2026**
**COI**	**None**

Comments to the Author – bmjopen-2025-112993

The study is interesting as it explores the lived experiences of anxiety related to breathlessness in patients with advanced chronic respiratory disease, who are receiving specialist palliative care.

However, there are some concerns that need to be clarified:

1. As the interviews were semi-structured, the content and themes of the interview guide (if used) should be described. In addition, the duration of the interviews should be described.

2. As a thematic analysis was used it would be useful to present a table of the analytic process with a few examples (i.e., from the meaning units to themes). This would strengthen the study's transparency.

3. Please consider revision of the result section. As the results are presented mostly through quotes, there is a lack of depth regarding the thematic analysis as presented.

4. The discussion would profit on revision with the focus of more depth regarding existing research in this field. As presented, the discussion may now be perceived as an extension of the result section.

VERSION 1 - AUTHOR RESPONSE

Reviewer: 1

Dr. Tracy Smith, Westmead Hospital, Faculty of Medicine and Health

Comments to the Authors:

The authors have undertaken a qualitative study of breathlessness and anxiety in patients with chronic respiratory disease. The methods are adequate to the research question and the analysis seems to be superficial, but reasonable.

However, the authors have not sufficiently engaged with the deep and long history of research in this area which goes back at least 40 years. The discussion, in particular, is underdeveloped and needs more work. Much of the discussion is a restatement of the results. Other than a brief mention of a paper from Bailey et al, the authors have not situated their paper within the broader literature. Perhaps due to this, it is not clear what the unique and new offering of this paper is to the literature. I think there may be some insights in the results about patients' understanding of the difference between breathlessness-induced anxiety and psychologically-based anxiety which may form the basis of a unique contribution. The experience of breathlessness and anxiety have been explored previously, though there is perhaps room to further explore this. Additionally, the discussion is missing at least some mention of implications for clinicians/ policy makers, as well as implications for future research.

Hence I recommend a major rewrite. The authors need to show evidence of an extensive review of the literature and develop and argument about the unique offering that this piece adds in the context of the available literature.

This may require:

Some specific comments:

• Note should be made of the SRQR reporting standards in the methods. SRQR checklist noted in the methods.

• Researchers characteristics are insufficiently described in the manuscript. Mention should be made at least of their discipline and other characteristics. Assuming the primary and secondary analysts are authors their initials should be included on page 6. Methods section expanded to describe researcher characteristics, including discipline, role in the analysis, and any prior relationship to participants.

• Authors note that techniques to improve trustworthiness are discussed on page 5. I cannot see this discussion on either page 5 or 6, but note used of techniques to enable credibility and confirmability on page 7. These contribute to trustworthiness but are not sufficient. There is no mention in particular of triangulation. Methods updated to include descriptions of trustworthiness and triangulation by primary analyst – including table of analytic process and examples.

• Trustworthiness does not appear to be specifically discussed in the limitations. Addressed as above.

• The conclusions drawn on page 15 lines 44 – 50 regarding suitability of a unified approach to care in breathlessness clinics is an over reach compared to the data presented and requires substantial revision. The discussion section has been re-written and this statement has been removed. The relevance of our results has been stated and linked to the relevant literature.

Reviewer: 2 [SEE ATTACHED FILE FOR COMMENTS FROM REVIEWER 2]

Dr. Vivi Lycke Christensen, University of South-Eastern Norway

Comments to the Authors:

The study is interesting as it explores the lived experiences of anxiety related to breathlessness in patients with advanced chronic respiratory disease, who are receiving specialist palliative care.

However, there are some concerns that need to be clarified:

1. As the interviews were semi-structured, the content and themes of the interview guide (if used) should be described. In addition, the duration of the interviews should be described. Topic guide updated and included as a supplementary file. Duration of interviews also described.

2. As a thematic analysis was used it would be useful to present a table of the analytic process with a few examples (i.e., from the meaning units to themes). This would strengthen the study's transparency. Table of analytic process and examples added to manuscript.

3. Please consider revision of the result section. As the results are presented mostly through quotes, there is a lack of depth regarding the thematic analysis as presented. Results section expanded by primary analyst and additional points added for each theme beyond direct quotations.

4. The discussion would profit on revision with the focus of more depth regarding existing research in this field. As presented, the discussion may now be perceived as an extension of the result section. Discussion section re-written and linked to current literature in the field.

VERSION 2 - REVIEW

**Reviewer**	**1**
**Name**	**Smith, Tracy**
**Affiliation**	**Westmead Hospital, Department of Respiratory and Sleep Medicine**
**Date**	**08-Mar-2026**
**COI**	

Thank you for asking me to re-review "Helps my anxiety because I'm managing my breathlessness": a qualitative exploration of anxiety and breathlessness in patients with advanced chronic respiratory disease receiving specialist palliative care. The manuscript is greatly improved from the previous version; however, I think that some further refinement is still required.

The abstract is generally well written; however, the last sentence in the conclusion is not supported by the data in the manuscript and should be withdrawn.

Introduction

There are some odd choices of references, and the authors may wish to update these. Particularly, reference 6 is somewhat older, and newer references are available. In addition, references 10 and 11 could be reviewed. While these are seminal references in the field, systematic reviews of breathlessness intervention services have been published since these trials were undertaken and would make better references here. For instance - Brighton LJ, Miller S, Farquhar M, Booth S, Yi D, Gao W, et al. Holistic services for people with advanced disease and chronic breathlessness: a systematic review and meta-analysis. Thorax. 2019;74(3):270-81. Or Spathis A, Reilly CC, Bausewein C, Reinke LF, Romero L, Smallwood NE, et al. Multicomponent services for symptoms in serious respiratory illness: a systematic review and meta-analysis. Eur Respir Rev. 2024;33(174).

In the introduction, and elsewhere in the paper, there is an implication that breathlessness clinics or breathlessness intervention services are only undertaken in specialist palliative care. This is erroneous. While most of these services are undertaken in specialist palliative care, there are certainly services that exist as part of respiratory services Smith TA, Roberts

MM, Luckett T, Cho JG, Klimkeit E, Stephenson H, McCaffrey N, Kirby A, Wheatley JR. Multidisciplinary, non-pharmacological breathlessness intervention service for patients with moderately severe to severe COPD: a randomised controlled trial. Thorax. 2025 Nov 27.

Methods

Regarding the methods, the first sentence does not read properly; I think a couple of words are missing, and this requires review.

In the first paragraph of the methods and the paragraph pertaining to participants, it is difficult to know any detail about the services from which the patients were recruited. Elsewhere in the manuscript, I gather that the patients recruited to this study had previously completed some sort of breathlessness intervention service. While the details may not be for all readers, I think it would be helpful to have an appendix that gives a sense of what breathlessness intervention patients had already received and where they are in this journey. Are patients at the beginning of this journey, or have they already been seeing a breathlessness intervention service and the specialist palliative care service for a period of time?

In terms of recruitment, it would be helpful to know the number of people contacted and the number that accepted the invitation.

The data collection section is greatly improved. However, knowing the mean duration of the interviews somewhere in the results would be helpful. I note that the data collection section suggests the interviews lasted between 30 to 45 minutes, but this is a little vague.

The data analysis section is significantly improved, and I am grateful to the authors for this significant improvement. I note that the SRQR guidelines mention the idea of triangulation in the techniques to enhance trustworthiness section. The word ‘triangulation’ is used in this manuscript, but the usage differs from the more common usage in qualitative research. More commonly, triangulation involves a third researcher who has a general overview of the transcripts but is not involved in the detailed analysis. The authors should state whether triangulation was or was not used, and if it was not, justify this choice.

I think the inclusion of Table 1 in the paper is a great improvement compared to the previous version of this manuscript. It significantly improves the reader’s understanding of the analysis undertaken.

Results

In the results section, the participants are described with a range of ages and their diagnoses. I think it would be useful to have a table showing this data along with results from the IPOS, which was used as an inclusion criterion for these patients. Additionally, some measure of their baseline breathlessness—either from the IPOS or the modified Medical Research Council Breathlessness Scale or something similar—would be helpful to contextualise the patients and give the reader an understanding of whether the patients they care for are similar to or different from those recruited for this study.

The results section is generally very well written, and the inclusion of quotes is very good, helping to illustrate the points the authors are making. There are significant areas of novelty in this results section. However, I am interested that there is no mention of the use of handheld fans as a measure for managing breathlessness. If the patients had completed a breathlessness intervention, this is a common mechanism for helping people manage their breathlessness, so I am interested that it has not come up.

Discussion

In terms of the discussion, I disagree with the authors' description of the breathing, thinking, functioning model. I do not think this model describes a vicious cycle of breathlessness. Rather, this model is a way of thinking about different components that might contribute to breathlessness and is more commonly used as such, rather than in the way the authors describe. This should be modified.

The second paragraph of the discussion includes suggestions of results which are not apparent in the results section and this needs to be remedied. Particularly, there is a mention that patients experience stigma surrounding visible signs of chronic respiratory disease, but I cannot see this in the results. Additionally, it is suggested in the discussion that many patients reported symptoms consistent with generalised anxiety disorder and none had received a formal diagnosis. This should appear in the results and not first appear in the discussion.

There is a sentence in the second paragraph of the discussion about psychological distress in family members. I cannot see that this is particularly linked to the surrounding text. Review is suggested.

The third paragraph of the discussion attempts to position the findings within the literature. Again, the choice of references here is odd: reference 25 is 10 years old, and reference 26 is 12 years old. There are certainly more recent references that could be included here. I think further literature review with more updated references would be helpful. Additionally, this further literature review might help the authors to better contextualise their findings. Overall the literature review which has contributed to the discussion appears a little superficial.

The fourth paragraph of the discussion again suggests that the holistic approach of multidisciplinary breathlessness services is within specialist palliative care, and this is not correct. Again, a broader literature review would have revealed this.

The third paragraph of the discussion suggests that patients had been through a breathlessness intervention service. This is not very clear from the methods and needs to be further clarified, as per my above comments.

I do think that the authors have a unique perspective here and could do more to build on this. KW is identified early in the manuscript as a senior qualitative researcher working primarily within mental health settings. Relatively few articles about breathlessness and anxiety have come from a mental health perspective, and I wonder if some further suggestions about future research in this area might be more developed in the discussion section.

The limitations section remains somewhat underdeveloped. Looking at the structure of the interview, there is a fairly long interview plan, and thus I imagine that breathlessness and anxiety did not feel like a particularly long part of the interviews, and this should at least be acknowledged as a limitation. Additionally, the interview plan starts by asking patients about the Alpha-Stim AID device and how they felt about this. It is at least a limitation to have discussed this before going on to discuss the patients' breathlessness and anxiety, and this should as a minimum be discussed as a limitation of the study, especially given the reported benefit of this device.

The discussion also does not have any implications for clinicians or implications for policy makers, which is a common but not universal component of a discussion section. I note that the SRQR does not suggest this as part of the discussion; however, BMJ guidelines have previously suggested this, and the authors might consider including such implications.

**Reviewer**	**2**
**Name**	**Christensen, Vivi Lycke**

Affiliation University of South-Eastern Norway, Department of Nursing and Health Sciences

Date 16-Mar-2026

COI

This paper holds the standard of publication.

VERSION 2 - AUTHOR RESPONSE

Reviewers comment	Response
The abstract is generally well written; however, the last sentence in the conclusion is not supported by the data in the manuscript and should be withdrawn.	The abstract conclusion has been redrafted to more accurately summarise the conclusions section of the main manuscript, with the last sentence being withdrawn as requested.
There are some odd choices of references, and the authors may wish to update these. Particularly, reference 6 is somewhat older, and newer references are available. In addition, references 10 and 11 could be reviewed. While these are seminal references in the field, systematic reviews of breathlessness intervention services have been published since these trials were undertaken and would make better references here. For instance - Brighton LJ, Miller S, Farquhar M, Booth S, Yi D, Gao W, et al. Holistic services for people with advanced disease and chronic breathlessness: a systematic review and meta-analysis. Thorax. 2019;74(3):270-81. Or Spathis A, Reilly CC, Bausewein C, Reinke LF, Romero L, Smallwood NE, et al. Multicomponent services for symptoms in serious respiratory illness: a systematic review and meta-analysis. Eur Respir Rev. 2024;33(174).	We thank the reviewer for the suggestion of references and have updated these accordingly.
In the introduction, and elsewhere in the paper, there is an implication that breathlessness clinics or breathlessness intervention services are only undertaken in specialist palliative care. This is erroneous. While most of these services are undertaken in specialist palliative care, there are certainly services that exist as	We have amended this statement in the introduction to read as below. We are grateful for the suggestion of the additional reference, which was published after this manuscript was originally submitted and has now been included.

Reviewers comment	Response
part of respiratory services Smith TA, Roberts MM, Luckett T, Cho JG, Klimkeit E, Stephenson H, McCaffrey N, Kirby A, Wheatley JR. Multidisciplinary, non-pharmacological breathlessness intervention service for patients with moderately severe to severe COPD: a randomised controlled trial. Thorax. 2025 Nov 27.	*Holistic multi-disciplinary breathlessness services, such as those offered by specialist palliative care or as part of some respiratory services, have demonstrated efficacy in supporting patients to manage their breathlessness.*
Regarding the methods, the first sentence does not read properly; I think a couple of words are missing, and this requires review.	The sentence has now been revised to improve readability and precision.
In the first paragraph of the methods and the paragraph pertaining to participants, it is difficult to know any detail about the services from which the patients were recruited. Elsewhere in the manuscript, I gather that the patients recruited to this study had previously completed some sort of breathlessness intervention service. While the details may not be for all readers, I think it would be helpful to have an appendix that gives a sense of what breathlessness intervention patients had already received and where they are in this journey. Are patients at the beginning of this journey, or have they already been seeing a breathlessness intervention service and the specialist palliative care service for a period of time?	We appreciate this information would be of benefit to some readers and thank the reviewer for the suggestion. Unfortunately, we do not have access to this data and therefore regret that we cannot include it in this paper.
In terms of recruitment, it would be helpful to know the number of people contacted and the number that accepted the invitation.	This qualitative analysis included only participants allocated to the intervention (Alpha-Stim) arm of the wider feasibility study. All participants in this group (n=11) were invited to take part in an interview, and all consented to participate. This has now been clarified in the Participants section of the Methods.
The data collection section is greatly improved. However, knowing the mean duration of the interviews somewhere in the results would be helpful. I note that the data collection section suggests the interviews lasted between 30 to 45 minutes, but this is a little vague.	This has been changed to give more detail and now reads:<br>*Interviews were audio-recorded onto an encrypted recorder interview lengths range from 28.25-38.59 minutes ($M_{minutes}$ = 32.59, SD = 3.54).*

Reviewers comment	Response
The data analysis section is significantly improved, and I am grateful to the authors for this significant improvement. I note that the SRQR guidelines mention the idea of triangulation in the techniques to enhance trustworthiness section. The word 'triangulation' is used in this manuscript, but the usage differs from the more common usage in qualitative research. More commonly, triangulation involves a third researcher who has a general overview of the transcripts but is not involved in the detailed analysis. The authors should state whether triangulation was or was not used, and if it was not, justify this choice.	We agree that triangulation is commonly understood as involving an additional independent researcher. We confirm that investigator triangulation was undertaken in this study. In addition to the two primary analysts, a third researcher (CF) reviewed the interview transcripts and the development of themes to provide an independent perspective on the analysis.<br>To avoid ambiguity, we have revised the manuscript to remove the previous reference to data source triangulation and to more clearly describe both the investigator triangulation process and how comparison across participants supported the analysis.
In the results section, the participants are described with a range of ages and their diagnoses. I think it would be useful to have a table showing this data along with results from the IPOS, which was used as an inclusion criterion for these patients. Additionally, some measure of their baseline breathlessness—either from the IPOS or the modified Medical Research Council Breathlessness Scale or something similar—would be helpful to contextualise the patients and give the reader an understanding of whether the patients they care for are similar to or different from those recruited for this study.	Thank you for this helpful suggestion. We agree that providing sufficient contextual information about participants is important for interpreting qualitative findings and assessing transferability.<br>Given the small sample size (n=11), we aimed to present key participant characteristics concisely within the text rather than as a separate table, in line with reporting conventions for qualitative studies. We have already included details on age range, gender, and primary diagnoses in the Results section.<br>All participants met the inclusion criterion of scoring >2 on both the 'shortness of breath' and 'anxious/worried' domains of the IPOS, indicating a clinically relevant level of symptom burden. To further clarify this, we have now added a brief statement in the manuscript to highlight that participants experienced moderate to high levels of breathlessness and anxiety at baseline.<br>While we recognise the potential value of additional tabulated data, we considered that inclusion of detailed individual-level scores (e.g., IPOS or mMRC) would add limited interpretive value in the context of this qualitative analysis and would be

Reviewers comment	Response
	disproportionate to the size and aims of the study.
The results section is generally very well written, and the inclusion of quotes is very good, helping to illustrate the points the authors are making. There are significant areas of novelty in this results section. However, I am interested that there is no mention of the use of handheld fans as a measure for managing breathlessness. If the patients had completed a breathlessness intervention, this is a common mechanism for helping people manage their breathlessness, so I am interested that it has not come up.	We agree that the use of handheld or electric fans is a well-recognised non-pharmacological strategy for managing breathlessness within breathlessness intervention services. A small number of participants in our study described using airflow (e.g., fans or open windows) to alleviate breathlessness and reduce anxiety. To address this, we have now included a brief reference and illustrative quote within the existing "Breathing exercises, distraction, and relaxation techniques" subtheme.
In terms of the discussion, I disagree with the authors' description of the breathing, thinking, functioning model. I do not think this model describes a vicious cycle of breathlessness. Rather, this model is a way of thinking about different components that might contribute to breathlessness and is more commonly used as such, rather than in the way the authors describe. This should be modified.	We have amended this section of the discussion to better contextualise the Breathing-Thinking-Functioning model with our findings, without comparisons to a vicious cycle.
The second paragraph of the discussion includes suggestions of results which are not apparent in the results section and this needs to be remedied. Particularly, there is a mention that patients experience stigma surrounding visible signs of chronic respiratory disease, but I cannot see this in the results. Additionally, it is suggested in the discussion that many patients reported symptoms consistent with generalised anxiety disorder and none had received a formal diagnosis. This should appear in the results and not first appear in the discussion.	The sentence regarding stigma has been reviewed. We believe in the results section we have highlighted the various components that may make up generalised anxiety disorder, including panic, fear, deep worry and physiological reactions. Participants describe having baseline generalised anxiety due to their diagnosis and prognosis. We then formulate these within the discussion section believe we have adequately described the components of an anxiety disorder within the results and are then formalising this in the discussion. However, for clarity we have added a sentence at the beginning of the results section as follows:<br>*None of our participants had a formal diagnosis of anxiety disorder.*

Reviewers comment	Response
There is a sentence in the second paragraph of the discussion about psychological distress in family members. I cannot see that this is particularly linked to the surrounding text. Review is suggested.	This has been removed.
The third paragraph of the discussion attempts to position the findings within the literature. Again, the choice of references here is odd: reference 25 is 10 years old, and reference 26 is 12 years old. There are certainly more recent references that could be included here. I think further literature review with more updated references would be helpful. Additionally, this further literature review might help the authors to better contextualise their findings. Overall the literature review which has contributed to the discussion appears a little superficial.	As above, we thank the reviewer for their suggestion of more recent references and have incorporated these into the discussion.
The third paragraph of the discussion suggests that patients had been through a breathlessness intervention service. This is not very clear from the methods and needs to be further clarified, as per my above comments.	We have amended this throughout the manuscript. The recruiting sites do offer holistic breathlessness intervention services, however as we are not provided specific information regarding this as to each participant's journey or involvement in the BIS, we have amended this section to focus more on the techniques participants have learned rather than their definite involvement in a breathlessness service.
Overall the literature review which has contributed to the discussion appears a little superficial.<br>The fourth paragraph of the discussion again suggests that the holistic approach of multidisciplinary breathlessness services is within specialist palliative care, and this is not correct. Again, a broader literature review would have revealed this.	We have amended this sentence to read:<br>*The findings reinforce the holistic approach offered by multidisciplinary BIS offered by a range of services.*
I do think that the authors have a unique perspective here and could do more to build on this. KW is identified early in the manuscript as a senior qualitative researcher working primarily within mental health settings. Relatively few articles about breathlessness and anxiety have come from a mental health perspective, and I wonder if some further suggestions about future	Thank you for this thoughtful and encouraging comment. We agree that applying a mental health perspective to the experience of breathlessness and anxiety offers valuable insights, and this is reflected in our interpretation of the findings, particularly in relation to the bidirectional and embodied nature of these symptoms. We have ensured that the implications for future research are reflected within the

Reviewers comment	Response
research in this area might be more developed in the discussion section.	existing text, particularly in relation to the need for further exploration of integrated psychological and symptom-focused approaches to care.
The limitations section remains somewhat underdeveloped. Looking at the structure of the interview, there is a fairly long interview plan, and thus I imagine that breathlessness and anxiety did not feel like a particularly long part of the interviews, and this should at least be acknowledged as a limitation. Additionally, the interview plan starts by asking patients about the Alpha-Stim AID device and how they felt about this. It is at least a limitation to have discussed this before going on to discuss the patients' breathlessness and anxiety, and this should as a minimum be discussed as a limitation of the study, especially given the reported benefit of this device.	We appreciate your comment regarding looking at this as a limitation, however, we would like to clarify that, although the interview topic guide included a section on the intervention as part of the wider feasibility study, the interviews were structured to ensure that participants' experiences of breathlessness and anxiety were explored in depth and formed a central focus of the discussion.<br>The ordering of topics did not prioritise discussion of the intervention over symptom experiences, and participants were able to provide detailed accounts of breathlessness and anxiety. As such, we do not consider this to have limited the depth or quality of the data generated for this analysis and have not included it as a limitation in the manuscript.
The discussion also does not have any implications for clinicians or implications for policy makers, which is a common but not universal component of a discussion section. I note that the SRQR does not suggest this as part of the discussion; however, BMJ guidelines have previously suggested this, and the authors might consider including such implications.	Towards the end of the discussion, we advise clinicians to specifically enquire with their patients regarding symptoms of anxiety and panic disorder, however we have added an additional statement following this to expand on clinical implications. We have chosen to not discuss any further implications as we do not wish to overstate our findings given the sample size and scope of this paper, based on previous reviewer feedback.
